# Pharmacological Characterization of Purified Full-Length Dopamine Transporter from *Drosophila melanogaster*

**DOI:** 10.3390/cells11233811

**Published:** 2022-11-28

**Authors:** Ciara Frances Pugh, Brian Thomas DeVree, Solveig Gaarde Schmidt, Claus Juul Loland

**Affiliations:** Laboratory for Membrane Protein Dynamics, Department of Neuroscience, Faculty of Health and Medical Sciences, University of Copenhagen, 2200 Copenhagen, Denmark

**Keywords:** dopamine transporter, protein purification, molecular pharmacology, scintillation proximity assay, radioligand, dopamine binding

## Abstract

The dopamine transporter (DAT) is a member of the neurotransmitter:sodium symporter (NSS) family, mediating the sodium-driven reuptake of dopamine from the extracellular space thereby terminating dopaminergic neurotransmission. Our current structural understanding of DAT is derived from the resolutions of DAT from *Drosophila melanogaster* (dDAT). Despite extensive structural studies of purified dDAT in complex with a variety of antidepressants, psychostimulants and its endogenous substrate, dopamine, the molecular pharmacology of purified, full length dDAT is yet to be elucidated. In this study, we functionally characterized purified, full length dDAT in detergent micelles using radioligand binding with the scintillation proximity assay. We elucidate the consequences of Na^+^ and Cl^−^ binding on [^3^H]nisoxetine affinity and use this to evaluate the binding profiles of substrates and inhibitors to the transporter. Additionally, the technique allowed us to directly determine a equilibrium binding affinity (K_d_) for [^3^H]dopamine to dDAT. To compare with a more native system, the affinities of specified monoamines and inhibitors was determined on dDAT, human DAT and human norepinephrine transporter expressed in COS-7 cells. With our gathered data, we established a pharmacological profile for purified, full length dDAT that will be useful for subsequent biophysical studies using dDAT as model protein for the mammalian NSS family of proteins.

## 1. Introduction

The dopaminergic system has a major involvement in many fundamental brain functions, such as movement, learning and reward [1]. Dopaminergic neurotransmission is spatially and temporally regulated by the dopamine transporter (DAT) through sodium ion-driven reuptake of dopamine from the extracellular space. Given this central role in regulation, DAT dysfunction has been linked to various neurological, psychiatric and neurodegenerative disorders, such as addiction, parkinsonism and attention deficit hyperactive disorder (ADHD) [2,3]. This association portrays DAT as a highly attractive therapeutic target, but likewise as a target for drugs of abuse, such as cocaine, amphetamines and cathinones [4,5]. More specifically, the addictive and rewarding effects associated with cocaine and other psychostimulants is hypothesized to stem from their interaction with the transporter [6,7,8,9].

DAT is a member of the solute carrier 6 (SLC6) family of human transporters—a subfamily of the larger neurotransmitter:sodium symporter (NSS) family, whose members span from prokaryotic species to human [10]. The closest human orthologues are the monoamine transporters for serotonin (SERT) and norepinephrine (NET) [3,11]. The majority of NSS members consist of 12 transmembrane domains (TMs) of mostly alpha-helical nature, connected by flexible extracellular and intracellular loops, with the N- and C-termini intracellularly located. TMs 1–5 and 6–10 are repeated with an inverted topological orientation, thereby forming a pseudo-symmetric structural repeat, known as the LeuT fold, as it was first observed in the crystal structure of its namesake, the leucine transporter (LeuT) from *Aquifex aeolicus* [12]. The first TM helix of each repeat is broken into two segments, where the high affinity primary substrate binding site (S1 site) lies. Here, the transporter’s substrate, as well as co-transported ions, are proposed to bind [12,13,14]. It is also thought to be the binding site for the majority of inhibitors targeting the monoamine transporters [15,16,17,18]. LeuT-fold proteins are considered to follow an alternating access transport mechanism, whereby the S1 site is alternatingly accessible to the extracellular and intracellular side of the membrane [19,20]. This is made possible through the dynamic movements of the external and internal gates of the transporter, allowing it to progress through its major conformational states in order to generate a transport cycle.

Presently, it has not been possible to purify human DAT (hDAT) as sufficiently stable and in quantities suitable for biophysical assays [21]. However, structural and functional studies of a homologue, the dopamine transporter from *Drosophila melanogaster* (dDAT), have been utilised to advance our understandings of hDAT’s molecular mechanisms [15,22,23,24]. dDAT displays high sequence homology with the SLC6 transporters (49, 52 and 45% for human DAT, NET and SERT, respectively) and approximately 80% similarity in TM domains. It is classified as a dopamine carrier because of its substrate specificity and restricted expression in dopaminergic neurons [25]. Crystal structures of a truncated and thermostabilized dDAT have been determined in complex with antidepressants, psychostimulants and its endogenous substrate, dopamine [14,15,24,26]. dDAT has a distinct pharmacological profile from hDAT. It maintains the substrate selectivity of DAT (dopamine > noradrenaline ≥ serotonin), however, it exhibits an inhibitory profile similar to NET [25]. Therefore, dDAT has a high affinity for tricyclic antidepressants and NET-selective compounds, such as nisoxetine. Altogether, dDAT’s high sequence identity and hybrid pharmacological profile renders it not only as a powerful tool for studying the pharmacology and substrate selectivity of DAT, but also other members of the NSS family [15].

The ability to express and purify dDAT provides a unique opportunity to investigate the structure-function relationships in these types of transporters with sophisticated biophysical methods. These include, but are not limited to x-ray crystallography, cryo-EM, NMR, hydrogen-deuterium exchange MS and fluorescent-based methods, either as an ensemble or in a single-molecule setup. Common for all applications, a detailed knowledge of the used ligand’s affinity is of outmost importance. The current available pharmacological profile for wild-type dDAT comes from transport inhibition studies of MDCK cells stably transfected with dDAT [25]. Presently, a pharmacological profile of the purified, full length dDAT is yet to be established.

Here, we assessed the molecular pharmacology of the purified, full length dDAT. This included the investigation of binding profiles of various substrates and inhibitors to purified dDAT through radioligand binding assays, to ultimately produce a pharmacological profile for purified dDAT. Additionally, we acquired a direct binding affinity for dopamine to dDAT, which is, to the best of our knowledge, the first report of this information in the literature. To assess the consequences of having dDAT in a detergent micelle, we furthermore investigated the binding of specific inhibitors and monoamines to dDAT expressed in COS-7 cells. The ligands’ affinities for human DAT and NET transfected into COS-7 cells was also determined. From this, we were able to directly compare the binding profile of purified dDAT to that of dDAT expressed in cells, as well as both human DAT and NET, and relate that to previous pharmacological studies on dDAT expressed in cell lines [25].

## 2. Materials and Methods

### 2.1. Constructs

Full length dDAT with a C-terminal thrombin cleavage site followed by a His_8_-tag was synthesized by GenScript Inc. and cloned into the pEG BacMam expression vector, which is suitable for both baculovirus generation and transient transfection into COS-7 cells, denoted as dDAT-His_8_.

Full length dDAT with a C-terminal thrombin cleavage site followed by a streptavidin binding peptide (SBP), TEV cleavage site and His_10_-tag was synthesized by GenScript Inc. and cloned into the pEG BacMam expression vector for generation of baculovirus, denoted as dDAT-SBP-His_10_.

Wild type hDAT and human NET (hNET) cloned into the pcDNA3.1 vector (ThermoFisher Scientific, Waltham, MA, USA) for transient transfection and expression in COS-7 cells.

### 2.2. Expression and Purification of dDAT

Full length dDAT with C-terminal His_8_ or with C-terminal SBP-His_10_ was expressed by transduction of Expi293F suspension cells (ThermoFisher Scientific, Waltham, MA, USA) with baculovirus produced in Sf9 cells (Expression Systems, Davis, CA, USA) and containing the pEG BacMam expression cassette with cloned dDAT cDNA [27]. After 48 h Expi293F cells were harvested by centrifugation (6000 × g) and membranes were prepared by homogenization with Dounce homogenizer in buffer (20 mM Tris, pH 8.0, 150 mM NaCl, 30% glycerol, 10 µg/mL benzamidine, 10 µg/mL leupeptin) and two rounds of sonication (Branson Sonifier 250 (Branson Ultrasonics, Brookfield, CT, USA), 50% duty cycle, power setting 2). Membranes were pelleted at 125,000 × g for 3 h at 4 °C. The pelleted membranes were homogenized in buffer (20 mM Tris, pH 8.0, 167 mM NaCl, 10 µg/mL benzamidine, 10 µg/mL leupeptin) with a Dounce homogenizer at a concentration of 5 mg total protein/mL. The homogenized membranes were solubilized by addition of concentrated detergent stock solution to make a solublization buffer (20 mM Tris, pH 8.0, 150 mM NaCl, 20 mM *n*-dodecyl β-D-maltoside (DDM), 4 mM cholesteryl hemisuccinate (CHS), 5 µg/mL benzamidine, and 10 µg/mL leupeptin) and incubated with gentle stirring for 1.5 h, followed by centrifugation for 30 min at 50,000 × g to pellet insoluble material. The supernatant was incubated with His-Pur Ni-NTA resin (Sigma Aldrich, St. Louis, MO, USA) for 2 h to bind detergent-solubilized dDAT. The resin was washed with buffer A (20 mM Tris, pH 8.0, 300 mM NaCl, 5% glycerol, 14 µM lipids (1-palmitoyl-2-oleoyl-sn-glycero-3-phosphocoline (POPC), 1-palmitoyl-2-oleoyl-sn-glycero-3-phosphoethanolamine (POPE), and 1-palmitoyl-2-oleoyl-sn-glycero-3-phospho-(1’-rac-glycerol) (POPG) at a weight ratio of 3:1:1), 1 mM DDM, 0.2 mM CHS, 5 µg/mL benzamidine, 10 µg/mL leupeptin) supplemented first with 30 and then with 60 mM imidazole on a gravity flow column. The protein was eluted from the affinity column with buffer A supplemented with 300 mM imidazole. The eluted protein was concentrated by ultrafiltration and run on a FPLC size exclusion column equilibrated in buffer A (Superdex^TM^ 200 10/300 GL, Cytiva, Marlborough, MA, USA) to increase purity and remove imidazole by buffer exchange. Finally, the protein was concentrated by ultrafiltration and stored at −80 °C for further use. All procedures were performed on ice or at 4 °C.

### 2.3. Radioligand Binding to Purified dDAT

Binding of substrates and inhibitors to purified dDAT was assessed through saturation and competition binding by scintillation proximity assay (SPA). Each assay was performed in white, clear-, flat-bottomed 96-well plates (Corning, Corning, NY, USA and Greiner Bio-One, Frickenhausen, Germany) and included either Copper HIS-Tag yttrium silicate (YSi) SPA beads (PerkinElmer, Waltham, MA, USA) when assessing binding in dDAT-His_8_, or Streptavidin YSi SPA beads (PerkinElmer, Waltham, MA, USA) when assessing binding in dDAT-SBP-His_10_, as well as a radioligand: [^3^H]nisoxetine (PerkinElmer, Waltham, MA, USA and Novandi Chemistry AB, Södertälje, Sweden), [^3^H]dopamine (PerkinElmer, Waltham, MA, USA) or [^3^H]mazindol (PerkinElmer, Waltham, MA, USA).

For competition binding, unless otherwise stated, 7 nM dDAT was mixed with SPA buffer (20 mM Tris-Cl (pH 8.0), 150 mM NaCl, 0.05% (*w*/*v*) DDM, 0.01% (*w*/*v*) CHS, 14 μM lipids (weight ratio of 3:1:1, POPC:POPE:POPG)) and 5% (*v*/*v*) SPA beads (25 mg/mL). Conditions using dopamine as the competing ligand also included 2 mM ascorbic acid to prevent oxidation of dopamine. This mixture was incubated on rotation for 45 min in darkness at 4 °C. The dDAT-SPA buffer mixture was added to the wells along with 100 nM of the desired tritiated ligand (often at 10% specific activity) and the dilution series of the unlabelled competitor. Non-specific binding was determined by adding 100 μM nortriptyline, to the dDAT-SPA buffer mixture and [^3^H]ligand. The plates were sealed, mixed at RT for 1.5 h on an agitator and left to settle at RT for 30 min. Counts per minute (c.p.m.) were measured on a 2450 MicroBeta^2^ microplate counter (PerkinElmer, Waltham, MA, USA). Plates were stored at 4 °C overnight and recounted to ensure that the original data was obtained at equilibrium. All experiments were performed in triplicates.

For saturation binding, unless otherwise stated, 7 nM dDAT was mixed with SPA buffer, 5% (*v*/*v*) SPA beads (25 mg/mL) and specified concentrations of [^3^H]nisoxetine in a titration series. Non-specific binding was determined by separately adding 100 μM nortriptyline to each [^3^H]nisoxetine condition with the dDAT-SPA buffer-beads mixture. The plate was incubated for 30 min on an agitator at RT and left to settle overnight at 4 °C. Counts per minute (c.p.m.) were measured on a 2450 MicroBeta2 microplate counter (PerkinElmer, Waltham, MA, USA). All experiments were performed in triplicates.

For ion binding studies, Na^+^-dependence of dDAT was determined using 7 nM of dDAT mixed with 120 nM [^3^H]nisoxetine (10% specific activity) and SPA buffer without NaCl (20mM Tris-HCl (pH 8.0), 0.05% (*w*/*v*) DDM, 0.01% (*w*/*v*) CHS, 14 μM lipids (weight ratio of 3:1:1, POPC:POPE:POPG)) supplemented with the indicated varying NaCl concentrations. Ionic strength was maintained by substituting NaCl with N-Methyl-D-glucamine (NMDG^+^) chloride. The indicated conditions were added and incubated on an agitator at 4°C for 1 h. 5% (*v*/*v*) Copper HIS-Tag YSi SPA beads (PerkinElmer, Waltham, MA, USA) was added to each well. Plates were sealed, mixed at RT on an agitator and left to settle at RT for 2 h. Counts per minute (c.p.m.) were measured on a 2450 MicroBeta2 microplate counter (PerkinElmer, Waltham, MA, USA). All experiments were performed in triplicates.

For [^3^H]nisoxetine saturation binding in varying ionic concentrations, 8.75 nM dDAT was preincubated in a 1.25 × solution consisting 6.25% (*v*/*v*) SPA beads in buffer containing the appropriate salt concentrations and all non-drug reagents for 45 min with agitation at 4 °C. 5 × dilutions of [^3^H]nisoxetine were added, bringing the final concentration of buffer components to 20mM Tris-HCl (pH 8.0), 0.05% (*w*/*v*) DDM, 0.01% (*w*/*v*) CHS, and 14 μM lipids (weight ratio of 3:1:1, POPC:POPE:POPG). The total concentration of all salts was 1000 mM, with sodium and chloride ion concentrations as given and the remaining salt concentration made up with acetate and NMDG^+^ as counterions. Plates were incubated further for 90 min at room temperature with shaking, the beads were allowed to settle at room temperature for 30 min, and the plate was measured on a 2450 MicroBeta^2^ microplate counter (PerkinElmer, Waltham, MA, USA). Non-specific binding was determined by adding a final concentration of 20 µM nortriptyline to the appropriate wells. Both total and non-specific binding measurements were run in quadruplicate with protein from the same purification batch to enhance comparability across conditions. In order to ensure that the assay’s B_max_ value was accurately determined, at least one of the three conditions that could be determined on each plate was a high Na^+^ condition.

### 2.4. Expression of Transporters in COS-7 Cells

COS-7 cells (a generous gift from Prof. U. Gether, Univ. of Copenhagen) were transiently transfected in Opti-MEM media (ThermoFisher Scientific, Waltham, MA, USA) using lipofectamine 3000 (ThermoFisher Scientific, Waltham, MA, USA) with 0.36 µg hDAT in the pcDNA3.1 vector, 0.36–0.47 µg hNET in the pcDNA3.1 vector or 0.83–1.6 µg dDAT in pEG BacMam vector per 10^6^ cells, in a lipofectamine:DNA (*w*/*w*) ratio of 3.5. Cells were seeded in 24-well plates (5 × 10^4^/well) coated with poly-ornithine and incubated in 10% CO_2_ at 37 °C in DMEM media supplemented with 1% FBS.

### 2.5. Competition Binding in COS-7 Cells

Competition binding assays were performed directly in the culture dish 48 h after transfection of the cells. The competition binding experiments were carried out at room temperature (RT). Prior to the experiment, each well was washed with 450 µL binding buffer at pH 7.4 containing 200 mM NaCl, 25 mM HEPES, 1.2 mM CaCl_2_, 1.2 mM MgSO_4_, 1 mM ascorbic acid, and 5 mM glucose. Binding was performed by addition of dilutions of unlabelled ligand together with 5.3 nM [^3^H]CFT (for hDAT, Novandi Chemistry AB, Södertälje, Sweden) or 3.1 nM [^3^H]nisoxetine (for hNET and dDAT, Novandi Chemistry AB, Södertälje, Sweden) in a total volume of 500 µL. After 2 h of incubation at room temperature, the buffer was removed and the cells were washed twice with 500 µL ice-cold binding buffer to remove unbound ligand. Cells were lysed in 250 µL of 1% sodium dodecyl sulfate (SDS) for 60 min at 37 °C, then transferred to 24-well sample plates (PerkinElmer, Waltham, MA, USA) and counted in a 2450 MicroBeta^2^ microplate counter (PerkinElmer, Waltham, MA, USA) after addition of 500 µL Opti-phase Hi Safe 3 scintillation fluid (PerkinElmer, Waltham, MA, USA). All experiments were performed in triplicates. Non-specific binding was determined in cells pre-incubated with either 10 µM nomifensine (for hDAT) or 100 µM nortriptyline (for hNET and dDAT).

### 2.6. Statistical Analyses

All statistical tests and data analyses were performed in GraphPad Prism 9 (GraphPad Software, Prism 9 version 9.2.0, San Diego, CA, USA). Unless otherwise specified, all data points are given as mean ± SEM (standard error of the mean) or mean [SEM interval]. Data obtained from competition binding analyses were fitted as a non-linear regression (curve fit) through an inhibitory dose–response model (variable slope), otherwise known as a four-parameter logistic equation. The non-specific binding condition was subtracted before fitting the data. From the logIC_50_, the IC_50_ can be obtained and, therefore, the affinity (K_i_) derived through the Cheng-Prusoff equation. For homologous competitive binding, whereby the same compound is used as both the labelled and unlabelled ligand, the Cheng-Prusoff equation can be converted to obtain the K_d_. Data plotted as a percentage of the control (absence of the competitive ligand) unless otherwise stated, i.e., normalized to the B_max_ determined from the sigmoidal fit. Saturation binding data were fitted after subtracting the non-specific binding from each condition. The data were fitted as a non-linear regression (curve fit) through a sigmoidal dose–response (variable slope) model to determine the K_d_. Ion binding study data were plotted using the same four-parameter logistic equation. For Figure S1, best fit estimators of the binding affinity are graphed along with the standard deviation for the parameter fit. The saturation curves on each plate were fitted with a globally shared B_max_ value to enable estimation of low affinity constants.

## 3. Results

### 3.1. Nisoxetine Binds to Purified dDAT in a Na^+^-dependent Manner

**Figure 1:**
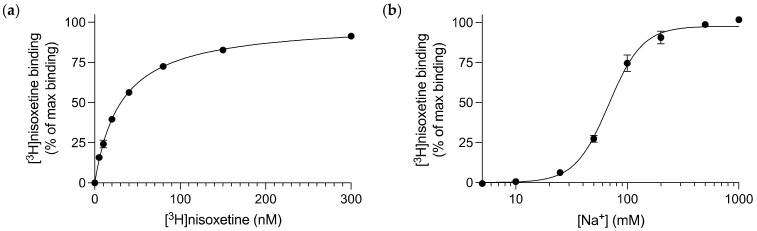
Characterizing [^3^H]nisoxetine binding to purified dDAT. (**a**) Saturation binding of [^3^H]nisoxetine to dDAT obtained a K_d_ of 31 [28; 34] nM for nisoxetine. Data are shown as mean [SEM interval], *n* = 3 from 3 independent dDAT purifications performed in triplicates and data are fitted as a one-site specific binding curve. (**b**) Na^+^-dependent [^3^H]nisoxetine binding to dDAT. The EC_50_ value for Na^+^ binding to dDAT in the presence of 120 nM [^3^H]nisoxetine was 68 [65; 71] mM. Ionic concentration is kept constant by substitution of Na^+^ with NMDG^+^. All data points represent mean ± SEM, *n* = 3 from at least 2 independent dDAT purifications performed in triplicates.

dDAT stably transfected into whole cells possesses a hybrid pharmacological profile, with an inhibitory profile more similar to hNET than hDAT [25]. In order to characterize the pharmacology of purified, full length, glycosylated dDAT, we first had to identify a high affinity, radiolabelled ligand to dDAT that could be used throughout this study for radioligand binding assays. The potent NET inhibitor, [^3^H]nisoxetine, was chosen as it is currently the best known, high affinity radiolabelled ligand available that binds to dDAT. The dDAT construct utilized for the following experiments was dDAT-His_8_. Using the scintillation proximity assay (SPA) [28], through saturation binding of [^3^H]nisoxetine on purified dDAT, a K_d_ of 31 [28; 34] nM (mean [SEM interval]) was determined for nisoxetine (Figure 1a). This high affinity radiolabelled ligand could then be utilized throughout subsequent binding assays as a competitive ligand, in order to understand the binding kinetics of other, unlabelled ligands.

Given that dDAT is a member of the NSS family and thereby symports sodium with each transport cycle, we assessed its affinity for sodium when binding [^3^H]nisoxetine. In a high concentration of [^3^H]nisoxetine (120 nM), we applied increasing Na^+^ concentrations and established that Na^+^ promotes [^3^H]nisoxetine binding to dDAT with an EC_50_ value of 68 [65; 71] mM (Figure 1b). The ionic concentration was kept constant throughout the experiment by substituting the change in Na^+^ concentration with N-methyl-D-glucamine (NMDG^+^). NMDG^+^ is a monovalent cation that does not support [^3^H]nisoxetine binding to dDAT [22].

**Figure 2:**
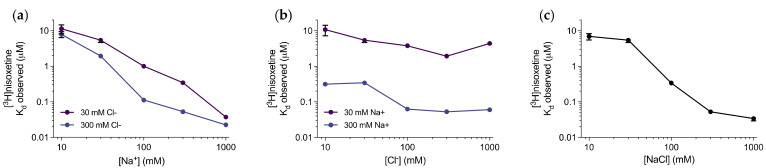
Influence of ions on the K_d_ for [^3^H]nisoxetine. (**a**–**c**) Log-log plots of fitted K_d_ values under varied or constant Na^+^ and Cl^−^ ion concentrations, as indicated on the graphs. (**a**) Na^+^-dependent changes in K_d_ for [^3^H]nisoxetine in the high (300 mM, blue) or low (30 mM, purple) Cl^−^ concentrations. (**b**) Cl^−^-dependent changes in K_d_ for [^3^H]nisoxetine in the high (300 mM, blue) or low (30 mM, purple) Na^+^ concentrations. (**c**) NaCl-dependent changes in K_d_ for [^3^H]nisoxetine. Each data point is graphed with the standard deviation for the fitted value, derived from [^3^H]nisoxetine saturation shown in Supplementary Figure S1. The data points are representative of triplicates from a singular dDAT purification, all plotted as mean ± SD.

To further investigate the ion binding properties of the transporter, we carried out saturation binding of [^3^H]nisoxetine on purified dDAT in varying ionic conditions (Figure S1). Specifically, we individually assessed the effect of differing both Na^+^ and Cl^−^ concentrations on the binding affinity of [^3^H]nisoxetine using NMDG^+^ as the counter-ion. From this dataset, it is clear that the applied Na^+^ concentration has a major impact on [^3^H]nisoxetine binding with an almost linear relationship between the Na^+^ concentration and increase in apparent [^3^H]nisoxetine affinity (K_d_), spanning more than 2 orders of magnitude between 10 and 1000 mM Na^+^ (Figure 2a). Conversely, the Cl^−^ concentration has a smaller effect on the K_d_ for [^3^H]nisoxetine binding with the most pronounced increase in [^3^H]nisoxetine affinity between 30 to 100 mM Cl^−^ in 300 mM Na^+^, thereafter, its effect appeared to be saturated. Under the low (30 mM) Na^+^ concentrations, the K_d_ for [^3^H]nisoxetine was largely independent of the Cl^−^ concentration (Figure 2b). When Na^+^ and Cl^−^ were added as a balanced salt, a more pronounced sigmoidal relationship between log(salt concentration) and log(K_d_) was still observed (Figure 2c). From this, we decided to obtain all subsequent binding data in 150 mM NaCl, leading to an observed K_d_ for [^3^H]nisoxetine in the low nanomolar range, and, most importantly, in an ionic concentration that is reasonably within the physiological range.

### 3.2. A Direct Binding Affinity for Dopamine to dDAT was Determined

**Figure 3:**
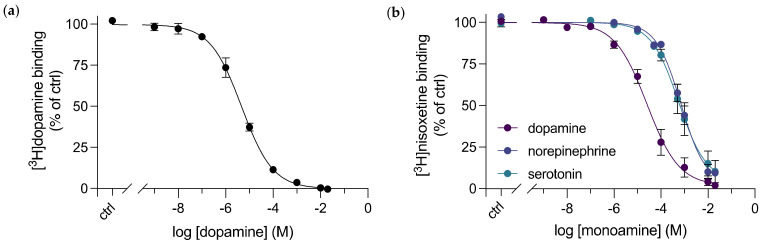
Binding of monoamine neurotransmitters to purified dDAT. (**a**) Homologous competitive binding of dopamine to purified dDAT. Dopamine K_d_ = 4.4 [3.4; 5.6] μM for dopamine binding to dDAT, *n* = 3 from 2 independent dDAT-SBP-His_10_ purifications, performed in triplicates. (**b**) Competitive inhibition of [^3^H]nisoxetine binding to purified dDAT by unlabelled dopamine (purple), norepinephrine (blue) and serotonin (green). The K_i_ values determined are as follows: 6.9 [4.5; 10.5] μM for dopamine, 184 [141; 239] μM for norepinephrine and 173 [97; 311] μM for serotonin. *n* = 3 from 2 independent dDAT-SBP-His_10_ purifications, performed in triplicates. Data are shown as mean ± SEM. ctrl (control): [^3^H]nisoxetine binding in the absence of the competitive ligand.

Previously, only indirect affinities of dopamine binding to DAT or other monoamine transporters have been measured through inhibition kinetics, for example, by inhibition of [^3^H]dopamine’s uptake in whole cells transfected with DAT [15,18,25,29], or by displacement of [^3^H]nisoxetine from purified dDAT with dopamine using SPA [14,23]. Dopamine is a more challenging ligand to work with as it has a known low affinity to the transporter and a consequently high dissociation rate, which does not allow for a washing step to remove unbound ligand. In addition, dopamine oxidizes readily in aqueous solutions at a physiological pH. In the context of SPA, the most commonly used SPA beads are Cu^2+^-coated, and therefore bind to His-tagged proteins, such as the dDAT-His_8_ construct used throughout this study. It is likely that dopamine interacts directly with the Cu^2+^-chelated beads resulting in an immense background signal. Cu^2+^ has also been shown to catalyze the oxidation process of dopamine making the assay unreliable [30,31]. Altogether, this may influence the signal from SPA, producing an inaccurate dose–response curve. To avoid Cu^2+^, we purified dDAT from a construct with a streptavidin binding peptide at its C-terminal end (dDAT-SBP-His_10_), in order to utilize the SBP-streptavidin interaction for SPA through streptavidin-coated SPA beads. Note that the SPA technology does not require a washing step. By homologous competitive [^3^H]dopamine binding to purified dDAT, we were able to measure the direct binding between dopamine and dDAT. A K_d_ for dopamine of 4.4 [3.4; 5.6] μM was determined (Figure 3a). The affinity is slightly higher than when measured as K_i_ for [^3^H]nisoxetine displacement or as K_m_ for [^3^H]dopamine uptake into intact COS-7 cells (Table 1).

### 3.3. Purified dDAT Displays Substrate Selectivity for Dopamine over Other Monoamine Neurotransmitters

A study assessing the pharmacological sensitivity of [^3^H]dopamine uptake in MDCK cells transfected with dDAT revealed a pharmacological profile for dDAT that was distinct from hDAT and most similar to the reported pharmacological profile in hNET [25]. To date, this is the only pharmacological profile available for dDAT and—in spite of extensive dDAT structural studies in complex with various ligands - a pharmacological profile for inhibitors and substrates of the purified, full length dDAT is yet to be elucidated. Through competitive inhibition of [^3^H]nisoxetine binding to purified dDAT by unlabelled substrates, we determined and compared the K_i_ for the endogenous human monoamine transporter substrates: dopamine, norepinephrine and serotonin (Figure 3b, Table 1). The inhibition constants we obtained, established a substrate selectivity that is consistent with what has been determined previously for the transporter in cells [25]: dopamine (K_i_ = 6.9 [4.5; 10.5] μM) > norepinephrine (K_i_ = 184 [141; 239] μM) and serotonin (K_i_ = 173 [97; 311] μM).

**Figure 4:**
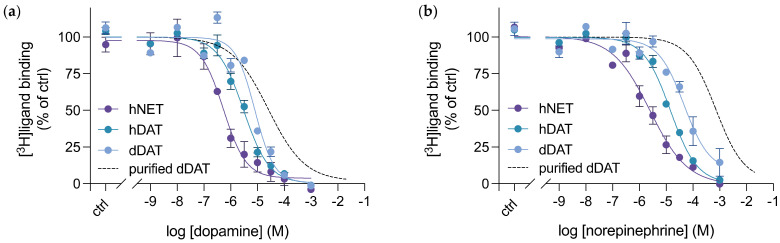
Competitive inhibition of radioligand binding to monoamine transporters in COS-7 cells by (**a**) dopamine and (**b**) norepinephrine. dDAT (blue circles), hDAT (green circles) and hNET (purple circles) were transiently transfected into COS-7 cells and underwent competitive radioligand binding. [^3^H]nisoxetine was used in the radioligand assays for both dDAT and hNET, and [^3^H]CFT was used in the radioligand assays for hDAT. The binding curves established are compared to dopamine and norepinephrine binding in purified dDAT (black dashed line), whose data curve comes from Figure 3b. The K_i_ values determined from these assays can be found in Table 1. All data points represent mean ± SEM, *n* = 3–5 from independent transfections of COS-7 cells. ctrl (control): [^3^H]ligand binding in the absence of the competitive substrate.

To determine the consequences associated with substrate affinities when having dDAT purified in a detergent micelle, we collected data to the binding profiles of dDAT expressed in whole cells. To also compare with affinities for hDAT and hNET, we expressed them in the same system. Thus, COS-7 cells were transfected to transiently express either dDAT, hDAT or hNET, and further used for transporter inhibition studies. Through competitive inhibition of [^3^H]nisoxetine binding or [^3^H]CFT (a cocaine analogue), for dDAT/hNET and hDAT, respectively, by the unlabelled endogenous substrates for each transporter, we obtained K_i_ values for dopamine and norepinephrine to each transporter (Figure 4). Similar K_i_ values (by inhibition of [^3^H]nisoxetine binding) were determined for dopamine binding to purified dDAT and dDAT in whole cells (Figure 4a). For norepinephrine binding, the change in K_i_ shows an approximate 2-times increase for purified dDAT (Figure 4b). Consistent with previous studies, both dopamine and norepinephrine bind hNET with high potency [36], obtaining K_i_ values of 430 nM and 1.8 μM, respectively. The inhibition constants determined for both monoamines to dDAT expressed in COS-7 cells were higher than the previously published IC_50_ values determined from transport inhibition studies with dDAT expressed in MDCK cells [25]. This could indicate that the transporter environment, here expressed by different cell lines, may influence its substrate binding kinetics. However, fundamental procedures, such as the expression vector and transfection method used also differed.

### 3.4. Purified dDAT Harbours an Inhibitory Profile Similar to hNET

In order to understand the inhibition profile for purified, full length, glycosylated dDAT in a detergent micelle, we performed competitive inhibition studies of [^3^H]nisoxetine binding to purified dDAT by unlabelled inhibitors. We determined the inhibition constants for 18 high affinity inhibitors of monoamine transporters and ranked them in order of potency (Table 1, Supplementary Figure S2). This list of inhibitors included selective serotonin reuptake inhibitors (SSRIs), tricyclic antidepressants (TCAs), norepinephrine reuptake inhibitors and both typical and atypical dopamine reuptake inhibitors, i.e., those that convey the stereotypical rewarding effects of DAT inhibition versus those that do not [34,37]. The potency ranking revealed a profile similar to what has been seen previously [25], whereby dDAT’s inhibitory profile resembles that of hNET. The NET-selective inhibitor, nisoxetine, topped the rankings. Among the top 6 most potent inhibitors, 4 were the TCAs: imipramine, amitriptyline, desipramine and nortriptyline. These are considered serotonin and norepinephrine reuptake inhibitors (SNRIs), thus further substantiating the NET-like inhibitor pharmacology of dDAT. The SSRIs: paroxetine, fluoxetine and S-citalopram, retained the order of potency observed in the literature for both hDAT and hNET [32,35]. The DAT-selective compounds, such as the classic cocaine-like DAT inhibitors, exhibited decreased affinities in dDAT compared to hDAT (Table 1, Supplementary Figure S2).

**Figure 5:**
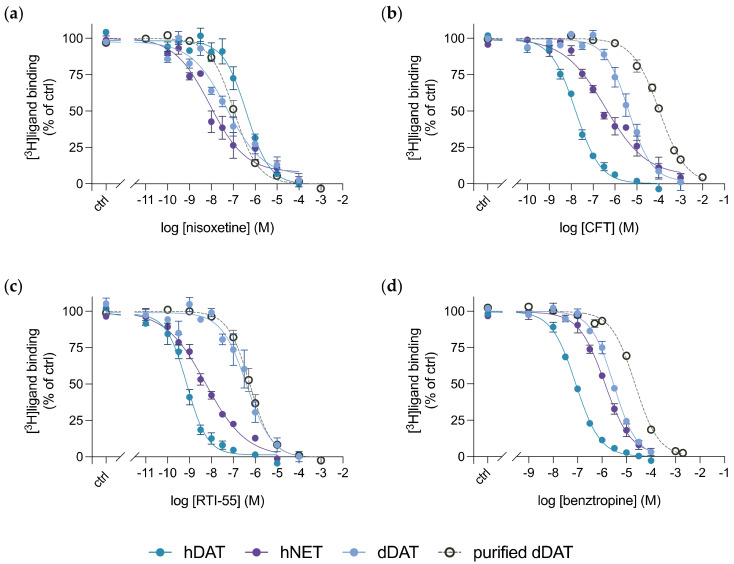
Competitive inhibition of radioligand binding to monoamine transporters in COS-7 cells or purified dDAT by (**a**) nisoxetine, (**b**) CFT, (**c**) RTI-55 and (**d**) benztropine. dDAT (blue filled circles), hDAT (green filled circles) and hNET (purple filled circles) were transiently transfected into COS-7 cells and underwent competitive radioligand binding. Purified dDAT (grey empty circles) also underwent competitive radioligand binding assays using SPA. [^3^H]nisoxetine was used in the radioligand assays for both dDAT and hNET, and [^3^H]CFT was used in the radioligand assays for hDAT. The K_i_ values determined from these assays can be found in Table 1. All data points represent mean ± SEM, *n* = 3–4 from independent transfections of COS-7 cells and *n* = 3 from at least 2 independent dDAT purifications performed in triplicates. ctrl (control): [^3^H]ligand binding in the absence of the competitive inhibitor.

Next, we compared the inhibition constants from purified dDAT with the binding potencies determined from dDAT, hDAT and hNET in whole cells, through competitive inhibition of the indicated radiolabelled ligands with selected unlabelled drugs (Figure 5). We chose to investigate 4 compounds: nisoxetine, CFT, RTI-55 and benztropine, in order to span from NET-selective to DAT-selective inhibitors, as well as including the atypical DAT inhibitor benztropine. A larger difference in binding affinity for the DAT-selective inhibitors, between purified dDAT in detergent micelles and dDAT expressed in whole cells is apparent. Both CFT (Figure 5b) and benztropine (Figure 5d) exhibited an approximate 6- and 2-fold decrease in affinity, respectively, for purified dDAT compared to dDAT in cells. However, binding of the cocaine analogue RTI-55 to dDAT does not change when evaluating binding of the purified transporter versus the transporter in COS-7 cells (Figure 5c). CFT and RTI-55 only differ by the substitution of the fluorine in CFT with an iodine in RTI-55, and dDAT crystal structures in complex with either CFT or RTI-55 do not reveal any difference in binding between the two drugs [14]. Additionally, the binding affinities obtained for nisoxetine to purified dDAT, and both dDAT and hNET in whole cells are similar, lying within each other’s margin of error (Figure 5a). The data suggest that the transporter environment does not solely determine this binding difference observed between purified dDAT and dDAT in whole cells. Perhaps, the type of inhibitor also influences whether a difference in binding affinities is observed between purified dDAT in a detergent micelle and dDAT expressed in whole cells.

### 3.5. Mazindol Displays Time-Dependent, Biphasic Binding Kinetics to dDAT

When comparing the K_i_ values determined for purified dDAT with the IC_50_ values obtained from transport inhibition studies with dDAT transfected into MDCK cells [25], there are two major differences of note: the affinities for bupropion and mazindol (Table 1).

Bupropion is a well-known antidepressant that inhibits the uptake of dopamine and norepinephrine. It is considered an atypical DAT inhibitor as it lacks the cocaine-like reinforcing effects of “typical” DAT inhibitors seen in humans [38]. The K_i_ of 7.5 [7.4; 7.7] μM obtained for bupropion, was almost 7-fold lower than the IC_50_ value determined previously from transport inhibition in dDAT-MDCK cells [25]. Interestingly, it was the only obtained K_i_ value from this study that was lower in purified dDAT compared to dDAT in whole cells. However, the plotted competition binding curve of [^3^H]nisoxetine displacement by bupropion did not display any prominent irregularities to further explore experimentally (Supplementary Figure S2k).

**Figure 6:**
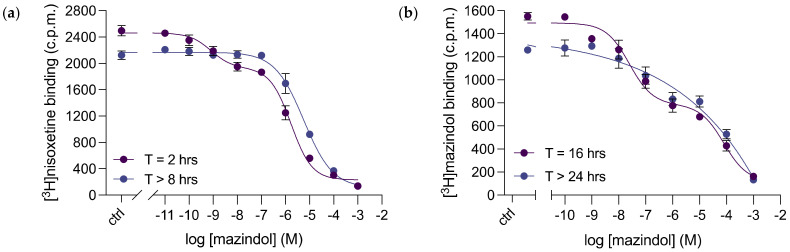
Mazindol binding to purified dDAT, assessed by radioligand binding. (**a**) Representative data of competitive inhibition of [^3^H]nisoxetine binding to purified dDAT by unlabelled mazindol. Data were collected after a 2 h incubation at RT or after a further incubation overnight at 4 °C, and fitted as two-site competition binding or as a sigmoidal dose–response curve (variable slope), respectively. K_i_ values for mazindol of 0.2 nM and 402 nM were estimated for the two binding populations in the displayed biphasic dataset for T = 2 h. Data points represent mean ± SEM performed in triplicates. The displayed monophasic binding curve of T > 8 h is representative data of *n* = 3 from 2 independent dDAT-His_8_ purifications, performed in triplicates. This acquired K_i_ for mazindol was 2.8 [2.2; 3.7] μM. Data points represent mean ± SEM. (**b**) Representative data of homologous competitive [^3^H]mazindol binding to purified dDAT. Data were collected after 8 h incubation at RT or after over 24 h incubation at 4 °C, and fitted as two-site competition binding or as a sigmoidal dose–response curve (variable slope), respectively. IC_50_ values for mazindol of 25 nM and 79 μM were estimated for the two binding populations in the displayed biphasic dataset for T = 16 h. Data points represent mean ± SEM performed in triplicates. The displayed monophasic binding curve of T > 24 h is representative data of *n* = 3 from 2 independent dDAT-His_8_ purifications, performed in triplicates. This acquired K_d_ for mazindol was 13 [5; 34] μM. Data points represent mean ± SEM. ctrl (control): [^3^H]ligand binding in the absence of the competitive inhibitor.

Mazindol is a tetracyclic compound that binds to all human monoamine transporters with high affinity and acts as an appetite suppressant for treatments against obesity [39], as well as a therapeutic for narcolepsy [40]. Remarkably, the acquired K_i_ of 2.8 [2.2; 3.7] μM for mazindol binding to purified dDAT was over 500-fold higher than what was estimated for dDAT in whole cells (Table 1). Focusing on its consequent competition binding curve, we observed mazindol binding to dDAT as biphasic (Figure 6a). When analyzed over time, the binding curve transformed to display monophasic binding kinetics without further deviations (Figure 6a)—resulting in the K_i_ value recorded in Table 1. To further assess mazindol binding to purified dDAT, we carried out homologous competitive [^3^H]mazindol binding to determine the direct binding affinity of mazindol to the transporter (Figure 6b). The acquired K_d_ for [^3^H]mazindol binding was 13 [5; 34] μM. Again, mazindol time-dependently bound in two populations to purified dDAT (Figure 6b)—biphasically, in higher and lower binding affinity populations at earlier timepoints, and monophasically, in the lower binding affinity population at later time points. However, the biphasic inhibition curve required a longer period of time to transform into a monophasic inhibition curve when examining homologous competitive [^3^H]mazindol binding (over 24 h) compared to competitive inhibition of [^3^H]nisoxetine binding (over 8 h). This discovery suggests that mazindol binding to dDAT is more complex than previously perceived.

## 4. Discussion

In this study, we have examined the impact of ions on ligand binding to purified dDAT, determined the direct binding affinity of the endogenous substrate, dopamine, to the transporter and established a pharmacological profile of inhibitor and substrate binding to purified dDAT. Additionally, we directly compared the binding profile of purified dDAT to that of dDAT in whole cells, as well as both hDAT and hNET, and from previous pharmacological studies.

Our data demonstrate the importance of Na^+^ and Cl^−^ ions to the binding of ligands to dDAT, and how [^3^H]nisoxetine affinity is highly dependent on Na^+^ concentration, with less effect of Cl^−^ concentration (Figure 2). This is consistent with the dDAT-nisoxetine crystal structure [26], where we see both sodium 1 and sodium 2 ion binding sites in close proximity (4 to 6 Å) to the ligand amine, likely allowing direct interaction of ligand and ion through hydrogen bonding or ion-dipole interactions.

The homologous competitive binding of dopamine to purified dDAT indicate that we established a technique to overcome the previous difficulties that arose when investigating direct dopamine binding to dDAT, i.e., high off-rate excluding a wash option to remove unbound dopamine, dopamine oxidation and the interaction with Cu^2+^ coated SPA beads. The K_d_ acquired for dopamine of 4.4 [3.4; 5.6] μM is similar to the K_i_ value determined from dDAT transfected into whole cells (7.7 [7.2; 8.3] μM), and the inhibition curve created did not display any obvious irregularities: monophasic, distinct maximum binding and a lower plateau that reached background count levels. Overall, validating the determined K_d_ for dopamine binding to purified, full length dDAT.

The determined dDAT binding profile demonstrated dopamine as the preferred monoamine neurotransmitter, reflected in the rank order of potency for monoamines-dopamine > norepinephrine/serotonin. This observed selectivity is similar to that seen in mammalian DATs and compatible with expression data from dDAT, which shows that dDAT mRNA expression in *Drosophila* is restricted to the dopaminergic neurons [25]. As nisoxetine and the TCAs: imipramine, amitriptyline, desipramine and nortriptyline, were determined to have the highest affinities for purified dDAT, its inhibitory profile is classified as NET-like. This hybrid pharmacological profile is also observed in dDAT stably transfected into whole cells and has been connected to the residues that interact with inhibitors in the binding pocket. Homology models analyzing these residues reported the binding pocket to be more closely related to the binding site of hNET than hDAT [26].

Before now, the only available pharmacological profile for full length dDAT came from transport inhibition studies of MDCK cells stably transfected with dDAT [25]. In Table 1, we compared the K_i_ values that we determined for purified dDAT with the K_i_ values estimated for dDAT in whole cells. The affinities for most of the tested compounds were lower for purified dDAT than dDAT in whole cells. These observed differences may stem from the transporter’s surrounding environment, as the detergent-solubilised protein is no longer embedded in the complex, heterogeneous, dynamic environment of a lipid bilayer. dDAT in a more native, cellular environment seems to favour the binding of these inhibitors and substrates, and it is likely that dDAT is more stable in the cellular membrane compared to a detergent micelle. Nevertheless, these observed differences in affinities between the purified transporter and transporter in whole cells were more apparent when comparing with the previous literature data from MDCK cells [25], than the data produced in-house using a COS-7 cell line. Both cell lines are derived from the kidney tissue of their respective species, although their morphologies differ as MDCK cells are epithelial, whilst COS-7 cells are fibroblast-like. The variation in binding affinities may be assay-specific, given that the previously determined pharmacological profile for dDAT acquired IC_50_ values from substrate transport inhibition of [^3^H]dopamine, whereas in this study, we determined a binding profile for dDAT through equilibrium binding inhibition of [^3^H]nisoxetine.

Through competitive inhibition of [^3^H]nisoxetine with mazindol, we discovered that mazindol bound time-dependently in two separate populations to purified dDAT, and similar results were observed for homologous competitive [^3^H]mazindol binding. It is likely that mazindol binds to the site occupied by nisoxetine, supposedly the primary binding site. However, it may bind this site from two transporter conformations, bringing about the two exhibited binding populations. The conformation that enables the stronger interaction between dDAT and mazindol appears to be less stable and converts slowly to the low-affinity binding conformation. Usually the low-energy, high affinity conformation will be preferred, whereas we see the opposite here as dDAT adopts the low-affinity state. Alternatively, mazindol binds to two distinct dDAT sites with the high affinity site being less stable. Binding of mazindol to dDAT could be likened to the binding of ibogaine to hSERT, which also seems to allow binding to two SERT conformations [41,42].

In this study, the established binding profile for purified, full length dDAT of monoamine transporter inhibitors and substrates creates an understanding of the transporter’s binding properties in a one-to-one system of purified protein to ligand binding, which can be used by other researchers to further elucidate dDAT’s, and other NSS members’ structural and functional properties. Additionally, we have acquired a direct binding affinity for dopamine to dDAT, an achievement that has not been met in literature before. Lastly, we have identified ligands, specifically mazindol, that may be of interest to use in further pharmacological studies of purified monoamine transporters.

## Figures and Tables

**Table 1:** Pharmacological characterization of purified dDAT and comparison with dDAT in whole cells (dDAT-COS and dDAT-MDCK) and other monoamine transporters. K_i_ values were determined from competitive inhibition of [^3^H]nisoxetine binding to purified dDAT. K_i_ values were determined from competitive inhibition of [^3^H]nisoxetine binding to dDAT and hNET in COS-7 cells or [^3^H]CFT binding to hDAT in COS-7 cells and compared to the IC_50_ values determined for dDAT transfected into MDCK cells (dDAT-MDCK; grey; *b*.) [25], as well as K_i_ values from the literature (cited below; light grey). ”DAT” and ”NET” represent mammalian DAT and NET as some of these compounds have not been investigated on the human monoamine transporters. Competitive inhibition curves for purified dDAT from which all K_i_ values were acquired can be found in Figure 1a, Figure 3b and Supplementary Figure S2. Competitive inhibition curves established in-house for dDAT, hDAT and hNET in whole cells can be found in Figure 4 and Figure 5. The IC_50_ values from dDAT-MDCK should be comparable to the K_i_ values as [^3^H]dopamine was used at concentrations < 10% of its respective K_m_ values. ^∗^ represents K_d_ values for [^3^H]ligand binding to purified dDAT or the monoamine transporters expressed in COS-7 cells determined by saturation binding or homologous competitive inhibition, respectively. All values in grey have been taken from previous literature. Data shown as means ± SEM or [SEM interval].

Compound	dDAT K_i_ (nM)	dDAT-COS K_i_ (nM)	dDAT-MDCK IC_50_ (nM)	DAT K_i_ (nM)	NET K_i_ (nM)
Nisoxetine	31 [28; 34]^*^	70 [42; 115] ^*^	5.6 ± 2.2 *^b^*	223 [154; 323]	8.8 [4.6; 15] ^*^
Imipramine	58 [51; 66]		30 ± 10 *^b^*	8500 ± 100 *^a^*	37 ± 2 *^a^*
Amitriptyline	97 [80; 117]		30 ± 1 *^b^*	3250 ± 20 *^a^*	35 ± 2 *^a^*
RTI-55	130 [95; 178]	299 [223; 400]	66 ± 10 *^b^*	0.5 [0.4; 0.63]	4 [3.3; 4.9]
Desipramine	141 [131; 151]		18 ± 5 *^b^*	3190 ± 40 *^a^*	0.83 ± 0.05 *^a^*
Nortriptyline	292 [255; 335]			1140 ± 30 *^a^*	4.37 ± 0.07 *^a^*
Paroxetine	322 [242; 428]		22 ± 4 *^b^*	490 ± 20 *^a^*	40 ± 2 *^a^*
Fluoxetine	512 [438; 599]		240 ± 60 *^b^*	3600 ± 100 *^a^*	240 ± 10 *^a^*
Mazindol	2800 [2200; 3700]		4.4 ± 2.2 *^b^*	8.1 ± 0.4 *^a^*	0.45 ± 0.03 *^a^*
JHW007	3100 [3000; 3300]			24.6 ± 8 *^c^*	1330 *^c^*
Benztropine	5200 [4800; 5700]	2800 [2600; 2900]		56 [54; 59]	989 [903; 1080]
Bupropion	7500 [7400; 7700]		48000 ± 5000 *^b^*	520 ± 20 *^a^*	52000 ± 1000 *^a^*
Cocaine	20000 [18000; 23000]		2660 ± 230 *^b^*	220 ± 9 *^a^*	1420 ± 50 *^a^*
S-citalopram	24000 [19000; 29000]		8100 ± 2100 *^b^*	27410 ± 3106 *^e^*	7841 ± 998 *^e^*
CFT	25000 [23000; 27000]	3900 [2800; 5400]		11 [9; 12] ^*^	357 [251; 508]
JJC8-088	33000 [30000; 35000]			2.53 ± 0.25 *^d^*	15000 ± 575 *^d^*
JJC8-091	199000 [188000; 211000]			289 ± 43 *^d^*	
Modafinil	554000 [516000; 596000]			2520 ± 204 *^d^*	> 100000 *^d^*
Dopamine	6900 [4500; 10500]	7700 [7200; 8300]	2900 ± 500 *^b^*	1900 [1600; 2200]	433 [343; 546]
Methamphetamine	13000 [10000; 16000]			2800 ± 100 *^a^*	660 ± 20 *^a^*
*D*-amphetamine	18000 [16000; 21000]		6600 ± 900 *^b^*	2900 ± 200 *^a^*	530 ± 40 *^a^*
Serotonin	173000 [97000; 311000]		43000 ± 7000 *^b^*	> 100000 *^a^*	> 100000 *^a^*
Norepinephrine	184000 [141000; 239000]	68000 [46000; 103000]	49000 ± 9000 *^b^*	9500 [8800; 10300]	1800 [1200; 2600]

a. Tatsumi et al. (1997). K_i_ values determined from radioligand binding assays with [^3^H]nisoxetine for hNET and [^3^H]CFT for hDAT transfected into HEK293 cells [32]; b. Pörzgen et al. (2001). IC_50_ values determined from uptake inhibition of [^3^H]dopamine for dDAT stably transfected into MDCK cells [25]; c. Agoston et al. (1997). K_i_ values determined from radioligand binding assays on membranes homogenised from rat caudate putamen with [^3^H]CFT for DAT and [^3^H]unknown ligand for NET [33]; d. Cao et al. (2016). K_i_ values determined from radioligand binding assays on membranes homogenised from rat caudate putamen (DAT) and frontal cortex (NET) with [^3^H]CFT for DAT and [^3^H]nisoextine for NET [34]; e. Owens et al. (2001). K_i_ values determined from radioligand binding assays with [^3^H]nisoxetine for hNET and [^125^I]RTI-55 for hDAT transfected into HEK293 cells [35].

## Data Availability

Draw data available upon request.

## Supplementary Materials

The following supporting information can be downloaded at: https://www.mdpi.com/article/10.3390/cells11233811/s1, Figure S1: Saturation binding of [^3^H]nisoxetine to purified dDAT-His_8_ under varied Na^+^ and Cl^−^ concentrations.; Figure S2: Pharmacological characterization of purified dDAT.
