# Supplementary material for: Pharmacological Characterization of Purified Full-Length Dopamine Transporter from Drosophila melanogaster
Source: Cells. 2022 Nov 28;11(23):3811. doi: 10.3390/cells11233811 (PMC9740255; doi:10.3390/cells11233811)
Supplement: Supplementary file 1 [file cells-11-03811-s001.zip › cells-2038502-supplementary.pdf]

Pharmacological Characterization of Purified Full-Length Dopamine Transporter from *Drosophila melanogaster*

Supplementary Figures:

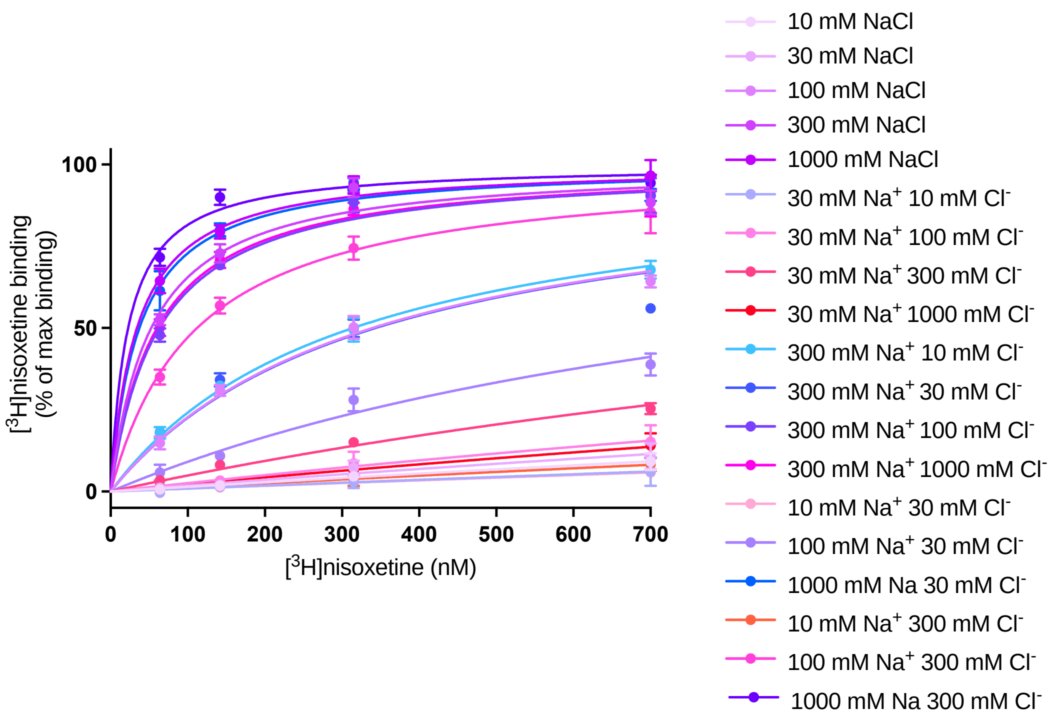

Supplementary Figure S1. Saturation binding of $[^3H]$nisoxetine to purified dDAT-His$_8$ under varied Na^+ and Cl^- concentrations. Assays were run with Cu^{2+} YSi SPA beads and non-linearly fitted with a one-site binding equation. Non-specific binding was determined by addition of 20 μM nortriptyline. Graphs are shown as relative binding, calculated with B_{max} values that were shared for each plate of 3 conditions, allowing the estimation of low affinity constants under low $[Na^+]$ conditions as long as at least one condition in each plate showed high affinity binding. Data are plotted as mean ± SD and data points are representative of triplicates from a singular dDAT purification.

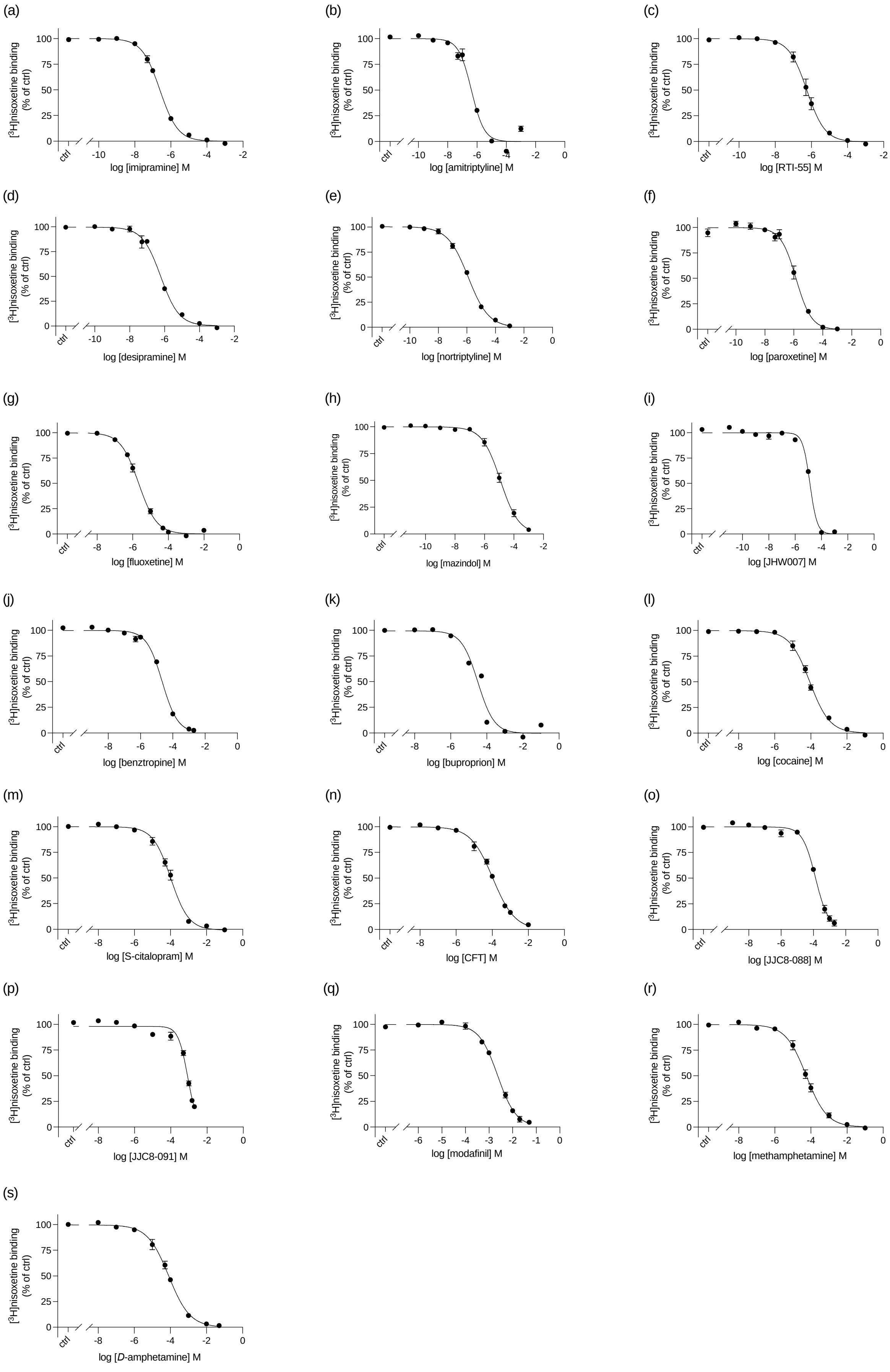

(a)
[3H]nisoxetine binding (% of ctrl)
ctrl
log [imipramine] M
(b)
log [amitriptyline] M
(c)
log [RTI-55] M
(d)
log [desipramine] M
(e)
log [nortriptyline] M
(f)
log [paroxetine] M
(g)
log [fluoxetine] M
(h)
log [mazindol] M
(i)
log [JHW007] M
(j)
log [benztropine] M
(k)
log [buproprion] M
(l)
log [cocaine] M
(m)
log [S-citalopram] M
(n)
log [CFT] M
(o)
log [JJC8-088] M
(p)
log [JJC8-091] M
(q)
log [modafinil] M
(r)
log [methamphetamine] M
(s)
log [D-amphetamine] M

Supplementary Figure S2. Pharmacological characterization of purified dDAT. Competitive inhibition curves of [^{3}H]nisoxetine binding to purified dDAT performed using Cu^{2+} YSi SPA beads. Competition binding outcompeting 100 nM [^{3}H]nisoxetine binding with (a) imipramine, (b) amitriptyline, (c) RTI-55, (d) desipramine, (e) nortriptyline, (f) paroxetine, (g) fluoxetine, (h) mazindol, (i) JHW007, (j) benztropine, (k) bupropion, (l) cocaine, (m) S-citalopram, (n) CFT, (o) JJC8-088, (p) JJC8-091, (q) modafinil, (r) methamphetamine, (s) *D*-amphetamine. Data are shown as mean ± SEM. All data are representative of n = 3 from 2 independent dDAT-His_8 purifications, performed in triplicates. ctrl (control): [^{3}H]nisoxetine binding in the absence of the competitive ligand.
